# A comprehensive analysis of the prognostic value and immune infiltration of low expression DBT in clear cell renal cell carcinoma

**DOI:** 10.3389/fphar.2022.1002588

**Published:** 2022-10-10

**Authors:** Wenjie Xie, Ping Xi, Yifu Liu, Zhicheng Zhang, Ting Sun

**Affiliations:** Department of Urology, The First Affiliated Hospital of Nanchang University, Nanchang, Jiangxi, China

**Keywords:** clear cell renal cell carcinoma, DBT, immune infiltration, prognostic biomarker, bioInformatic

## Abstract

**Background:** Although DBT is strongly associated with human tumorigenesis and progression through a variety of pathways, the role of DBT in clear cell renal cell carcinoma (ccRCC) has not been well established.

**Materials and methods:** The Cancer Genome Atlas (TCGA)-Kidney renal clear cell carcinoma (KIRC) databset provides RNA sequencing data and clinicopathological information on ccRCC. The Gene Expression Omnibus (GEO) database was used to validate the DBT expression levels, and qPCR was used to examine the DBT expression in renal cancer cell lines and ccRCC tissue samples from our centre. In parallel, DBT protein expression was explored in the Human Protein Atlas (HPA) database, and western blotting and immunohistochemistry of renal cancer cell lines and ccRCC tissues validated the results. Additionally, the diagnostic and prognostic value of DBT was comprehensively evaluated by receiver operating characteristic (ROC) curves, univariate and multivariate Cox regression analyses, and Kaplan‒Meier survival analysis. The protein‒protein interaction (PPI) network based on the STRING website, Gene Ontology (GO) analysis, Kyoto Gene and Genome Encyclopedia (KEGG) analysis and gene set enrichment analysis (GSEA) further provided a landscape of the molecular mechanisms of DBT in ccRCC. Finally, the TIMER 2.0, GEPIA and TISIDB websites were used to understand the relationship between DBT and immune characteristics.

**Results:** The mRNA expression and protein expression of DBT were significantly downregulated in ccRCC tissues relative to normal tissues, which was associated with poor clinical outcomes. DBT has an encouraging discriminatory power for ccRCC and is an independent prognostic factor for ccRCC patients. Mechanistically, DBT is mainly involved in the regulation of immune-related signalling pathways in ccRCC; it is associated with a variety of immune infiltrating cells and immune checkpoints.

**Conclusion:** DBT is a tumour suppressor gene in ccRCC and could be used as a new biomarker for diagnostic and prognostic purposes, and it is associated with immune infiltration in ccRCC.

## Introduction

Renal cancer is one of the most common urological tumours in the urinary system, ranking 6th and 9th in incidence among men and women in the United States, respectively (Siegel et al., 2022). The numbers of cancer cases and cancer-related deaths in China are expected to reach 77,410 and 46,345, respectively, in 2022 (Xia et al., 2022). Over 90% of renal cancers are renal cell carcinomas, mainly originating from the renal epithelium, with clear cell renal cell carcinomas (ccRCCs) being the most common subtype (Jonasch et al., 2014). Patients with ccRCC often lack typical clinical symptoms, resulting in a significant proportion of patients developing distant metastases by the time of their initial presentation (Grimaldo-Roque et al., 2022). In addition, although partial nephrectomy or radical nephrectomy can achieve good results for patients with early-stage renal cancer, one-third of patients still experience postoperative recurrence and metastasis (Stewart et al., 2014). The poor prognosis of this disease is highlighted by a 5-year survival rate of less than 10% in patients with advanced ccRCC (Choueiri and Motzer, 2017). In recent years, although antiangiogenic therapy and immune checkpoint inhibitors have brought satisfactory results for a proportion of ccRCC patients, the applicability and durability of treatment strategies remains a great challenge (Sanmamed and Chen, 2018). As a result, the molecular landscape of ccRCC needs to be continuously refined to achieve precise individualized treatment and more durable responses.

Dihydrolipoamide branched-chain transacylase E2 (DBT) is the transacylase subunit of the BCKDH complex; it is a key enzyme in amino acid metabolism, according to previous studies, and the main function of the BCKDH complex is to participate in the catabolic metabolism of branched-chain amino acids (Pettit et al., 1978; Hu et al., 1986). It has been shown that the interaction of DBT with peroxiredoxin V is involved in the response to hypoxic stress in the kidney (Ahn et al., 2015). In addition, mutations in the DBT gene can occur, and such mutations can cause maple syrup urine disease (MSUD) and may have serious consequences, such as dystonia or even death (Strauss et al., 2010). DBT is also the E2 subunit of the pyruvate dehydrogenase complex (PDC) (Tsvetkov et al., 2022), which is mainly involved in the TCA cycle and glycolytic pathways and has been shown by studies to be closely associated with the development and progression of cancer (McFate et al., 2008). The inhibition of PDC activity helps to reduce the malignant phenotype of human head and neck squamous cell carcinoma and inhibit the growth of colorectal cancer (McFate et al., 2008; Jin et al., 2020). It has been shown that there is specificity between DBT and signal transducer and activator of transcription 5 (STAT5) in leukaemic T-cell lines and that STAT5 can be translocated to mitochondria by cytokine regulation and tyrosine phosphorylation to bind to DNA and promote cancer cell growth through a cellular metabolic pathway (Chueh et al., 2010). Overall, this previous evidence suggests that DBT may play a specific role in human cancers.

However, the exact mechanism of action of DBT in ccRCC is still not well understood. Therefore, the purpose of this study was to investigate the expression level and prognostic value of DBT in ccRCC and to explore its potential mechanisms.

## Materials and methods

### Ethical statement

The study was conducted in accordance with the Declaration of Helsinki, and the protocol was approved by the Ethics Committee of the First Affiliated Hospital of Nanchang University Ethics number: (2021) Medical Research Ethics No. (12-024); date of approval 1 December 2021. We also selected 14 pairs of fresh ccRCC and adjacent nontumor tissues from our study centre. The use of these samples was approved by the ethics committee, and informed consent was obtained from all enrolled patients.

### Data acquisition and mining

The RNA sequencing (RNA-seq) data of ccRCC samples (539 tumour samples and 72 normal tissues)and corresponding clinical data were downloaded from The Cancer Genome Atlas (TCGA) database (https://portal.gdc.cancer. gov/). Gene Expression Omnibus (GEO) datasets (GSE53757 containing 72 matched pairs of renal clear cell carcinoma and normal kidney tissues, GSE53000 containing 56 renal clear cell carcinoma tissues and 6 normal kidney tissues, GSE40435 containing 101 matched pairs of renal clear cell carcinoma and normal kidney tissues, and GSE105261 containing 35 renal clear cell carcinoma tissues and 9 normal kidney tissues) from the National Center for Biotechnology Information (NCBI) (https://www.ncbi.nlm.nih.gov/gds/) were also included in this study for differential expression analysis of DBT (Clough and Barrett, 2016). The “ggplot” package of R software (v.3.6.3) was used to analyse the expression of DBT in ccRCC in the TCGA-KIRC and GEO databases. The Human Protein Atlas (HPA) (https://www.proteinatlas.org/) and the Clinical Proteomic Tumor Analysis Consortium (CPTAC) from UALCAN (http://ualcan.path.uab.edu/index.html)also provided data on the protein expression of DBT in ccRCC and normal tissues as well as relevant histological profiles for our study (Chandrashekar et al., 2017; Uhlen et al., 2017; Chandrashekar et al., 2022).

### Cell lines

HK-2, A498, 786-O, ACHN, Caki-1, and OSRC-2 cells were purchased from the Chinese Academy of Sciences (Shanghai, China). Cells were cultured in high-glucose DMEM (Solarbio, Beijing Solarbio Science & Technology Co., Ltd.),MEM (Boster, China) and 1,640 (Gibco, Carlsbad, CA) medium containing 10% FBS(VivaCell Biosciences Co., Ltd.) and 1% streptomycin-penicillin, and maintained at 37°C and 5% CO2.

### RNA extraction and quantitative real-time polymerase chain reaction

RNA was extracted from renal cancer cell lines (A498, 786-O, ACHN, Caki-1, and OSRC-2), a normal renal tubular epithelial cell line (HK-2) and 14 paired ccRCC tissues from our research centre by using TRIzol reagent (Cwbio, China) and NanoDrop 2000 software to determine the quality and concentration of RNA. A reverse transcription kit (TransGen Biotech, China, Beijing) was used to synthesize relative cDNA. qPCR was performed using the SYBR Real-Time PCR Kit (TransGen Biotech, China, Beijing), and the relative quantitative data were analysed using the 2^-ΔΔCt^ method. The forward primer sequence for DBT was 5′-TGG​TGC​TAC​AAT​GTC​ACG​CT-3′, and the reverse sequence was 5′-GCT​GGC​ACA​GCT​AGG​GTT​TA-3'. The reference gene was *β*-actin, in which the forward sequence was 5′-TCT​CCC​AAG​TCC​ACA​CAG​G-3′ and the reverse sequence was 5′-GGC​ACG​AAG​GCT​CAT​CA-3'.

### Western blotting

RIRA lysate buff was used to extract total protein from renal cancer cell lines (A498, 786-O, ACHN, Caki-1, and OSRC-2) and normal renal tubular epithelial cells (HK-2), and four pairs of ccRCC tissues and matched adjacent normal tissues from our study centre. Protein concentrations were determined by the BCA method using a bicinchoninic acid kit (CoWin Biosciences). Then, 30ug of total protein was separated by 10% SDS-PAGE (Servicebio, China) and transferred to PVDF membranes (0.45 mm,Immobilon-P Transfer Membrane). The membrane was subsequently blocked for 1 h at room temperature using 5% skim milk and inclubated with corresponding DBT antibody (Affinity Biosciences,DF13569,Rat, 1:1,000) or GAPDH antibody (ZSGB-BIO,TA-08,Mouse, 1:1,000) overnight at 4°C. After rinsing with TBST (Servicebio, China), the membrane was placed in the corresponding secondary antibodies and incubated for 1 h at room temperature, and rinsed again with TBST (Servicebio, China), and target protein expression levels were detected with an ECL kit (Fdbio science, China,Hangzhou) and a DNR Bioimaging System, analysed by using GraphPad Prism 9.

### Immunohistochemistry

First, 2 pairs of renal clear cell carcinoma and paraneoplastic normal tissue samples from our study centre were fixed with 10% formalin, dehydrated in a series of graded concentrations of ethanol, xylene transparent, waxed, embedded, and finally made into 5 µm sections. Before using the sections, they were dewaxed using xylene, hydrated in a gradient of ethanol, and incubated in 0.3% methanol-H_2_O_2_ solution for 30 min at room temperature to inactivate endogenous peroxidase. The sections were then boiled with 0.01 mol/L citrate buffer in the microwave for antigen repair, blocked in secondary antibody-derived nonimmune serum for 30 min, incubated overnight at 4°C with anti-DBT, washed in PBS, and incubated in secondary antibody for 1 h. The tissue sections were stained with diaminobenzidine and restained with haematoxylin, and the images were taken under a Zeiss microscope.

### Analysis of the prognostic value of dihydrolipoamide branched-chain transacylase E2

To further investigate the prognostic ability of DBT expression in ccRCC, the Mann‒Whitney *U* test of the “ggplot” package was used to explore the association between different clinical stages or states and DBT expression. Kaplan‒Meier (KM) survival curves drawn with the “survminer” and “survivor” packages of R software and receiver operating characteristic (ROC) curves drawn with the “pROC” package demonstrated the ability of high and low DBT expression to determine the prognosis of ccRCC patients with different clinical statuses.

### Univariate and multivariate cox regression analyses、construction of the nomogram and generation of calibration plots

Based on univariate and multivariate Cox regression analyses, we evaluated the relationship between DBT and relevant clinical features for prognostic evaluation of the overall survival (OS), disease-specific survival (DSS), and progression-free interval (PFI) of ccRCC patients by using the “survival” package of R software. A nomogram was constructed to determine the prognosis of patients based on DBT expression in the TCGA-KIRC database and relevant clinical data (age, T stage, N stage, M stage, pathological grade, histological grade, etc.) To validate its predictive ability, the “Caret” R package randomised the TCGA-KIRC dataset into training and test groups. The ROC curves and calibration plots at 1, 3 and 5 years further demonstrated the ability of the model to determine the prognosis of ccRCC patients.

### Protein–protein interaction and functional enrichment analyses

The extensive PPI data from the STRING website (https://string-db.org/) were used to analyse genes with some association with DBT; the PPI network was visualised using Cytoscape v3.9.1, and the associations between DBT and these genes were further validated (Szklarczyk et al., 2011). The TCGA-KIRC dataset was analysed for differential genes associated with DBT expression; 500 upregulated and 500 downregulated differential genes were screened and subjected to Gene Ontology (GO) enrichment analysis of biological processe (BP), cellular component (CC), and molecular function (MF), and Kyoto Encyclopedia of Genes and Genomes (KEGG) analysis with adjusted *p* < 0.05 and |log2 (fold change)| >1 as the thresholds (Yu et al., 2012). The weighted set cover approach was used to reduce redundancy and set the minimum number of genes to 10 and the number of simulations to 1,000; the “limma,” “org.Hs.eg.db,” “clusterProfiler” and “enrichplot” R packages were used in this analysis. In particular, to further explore the potential signalling pathways of DBT, gene set enrichment analysis (GSEA) was performed on these associated genes (Subramanian et al., 2005).

### TIMER 2.0, GEPIA and TISIDB

The correlations between DBT and tumour-infiltrating lymphocytes (TILs) in ccRCC were assessed using R software and TIMER 2.0 (http://timer.cistrome.org/) (Li et al., 2020). The correlations were further explored in terms of the expression of specific markers associated with DBT and TILs based on the “correlation” function of the GEPIA website (http://gepia2.cancer-pku.cn/). In addition, the TISIDB website (http://cis.hku.hk/TISIDB/) was used to explore correlations between DBT and immune checkpoint genes in ccRCC (Ru et al., 2019).

### Statistical analysis

All statistics were taken from the website corresponding to R software (v.3.6.3) (Kruppa and Jung, 2017) and the GEO2R website (https://www.ncbi.nlm.nih.gov/geo/geo2r/) (Davis and Meltzer, 2007), with some with special instructions for analysis from the corresponding website. Statistical analysis of the qPCR and WB results was performed by GraphPad Prism 9.0.0. The Wilcoxon signed rank test and Wilcoxon rank sum test were used to compare gene expression differences between normal and cancerous tissues. KM analyses, Cox proportional hazards models, and log-rank tests were used to determine the diagnostic and prognostic value of DBT in patients with ccRCC, and comparison of the correlation between two variables was performed by Spearman correlation analysis. All *p* values were corrected by the Benjamini‒Hochberg (BH) method, and a *p* value < 0.05 was considered statistically significant.

## Results

### Low expression of dihydrolipoamide branched chain transacylase E2 in clear cell renal cell carcinoma

**FIGURE 1:**
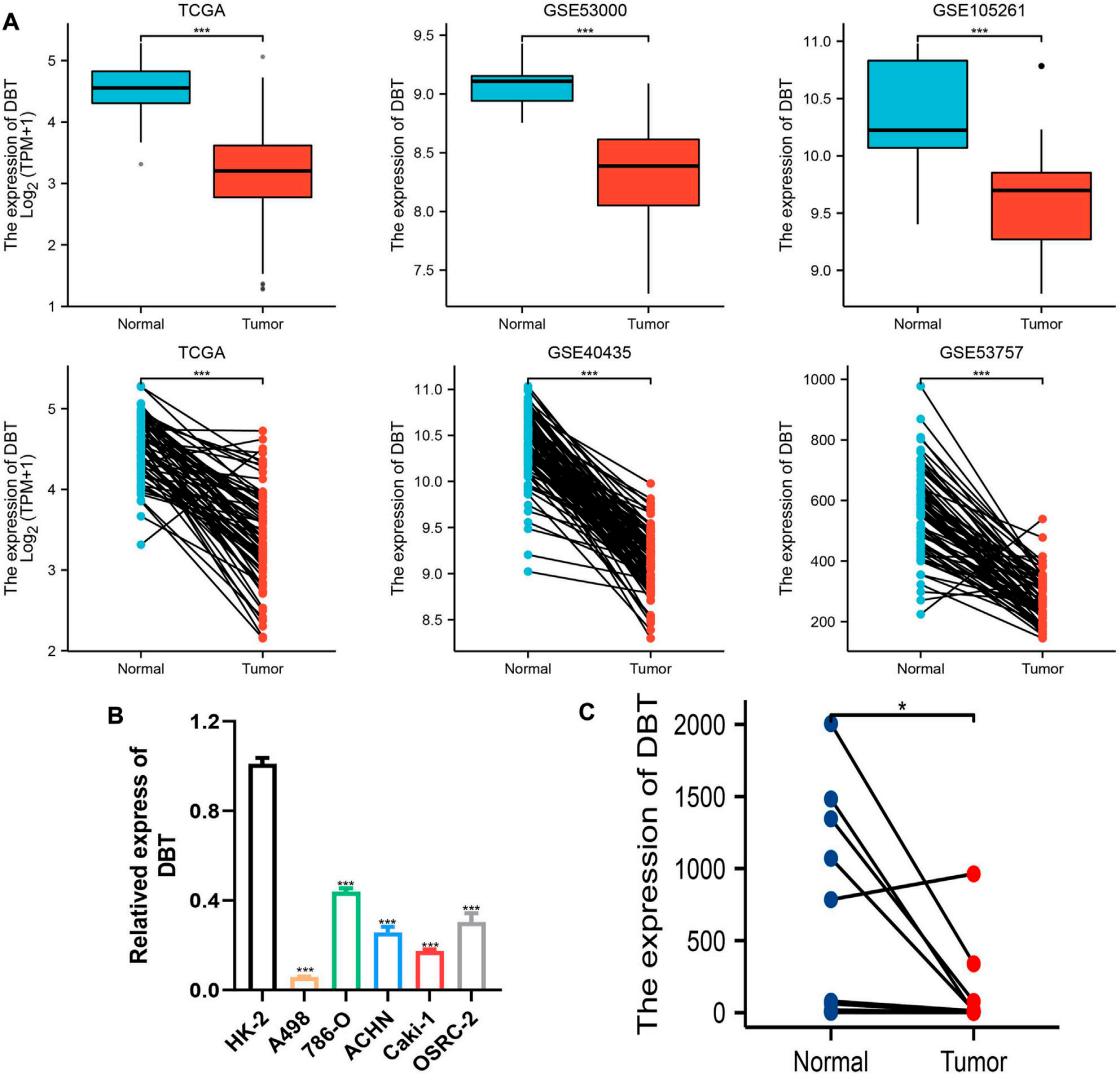
Low expression of DBT at the RNA level in ccRCC. **(A)** DBT showed low expression in the TCGA database and GSE40435, GSE5300, GSE53757, and GSE105261. **(B)** DBT was verified in renal cancer cell lines (A498, 786-O, ACHN, Caki-1, and OSRC-2) and renal tubular epithelial cells (HK-2) at the RNA expression level to have a low expression status. **(C)** DBT expression was lower in 14 renal clear cell carcinoma tissues than in the corresponding normal tissues. *p < 0.05, **p < 0.01, ***p < 0.001, “ns”: not statistically significant.

**FIGURE 2:**
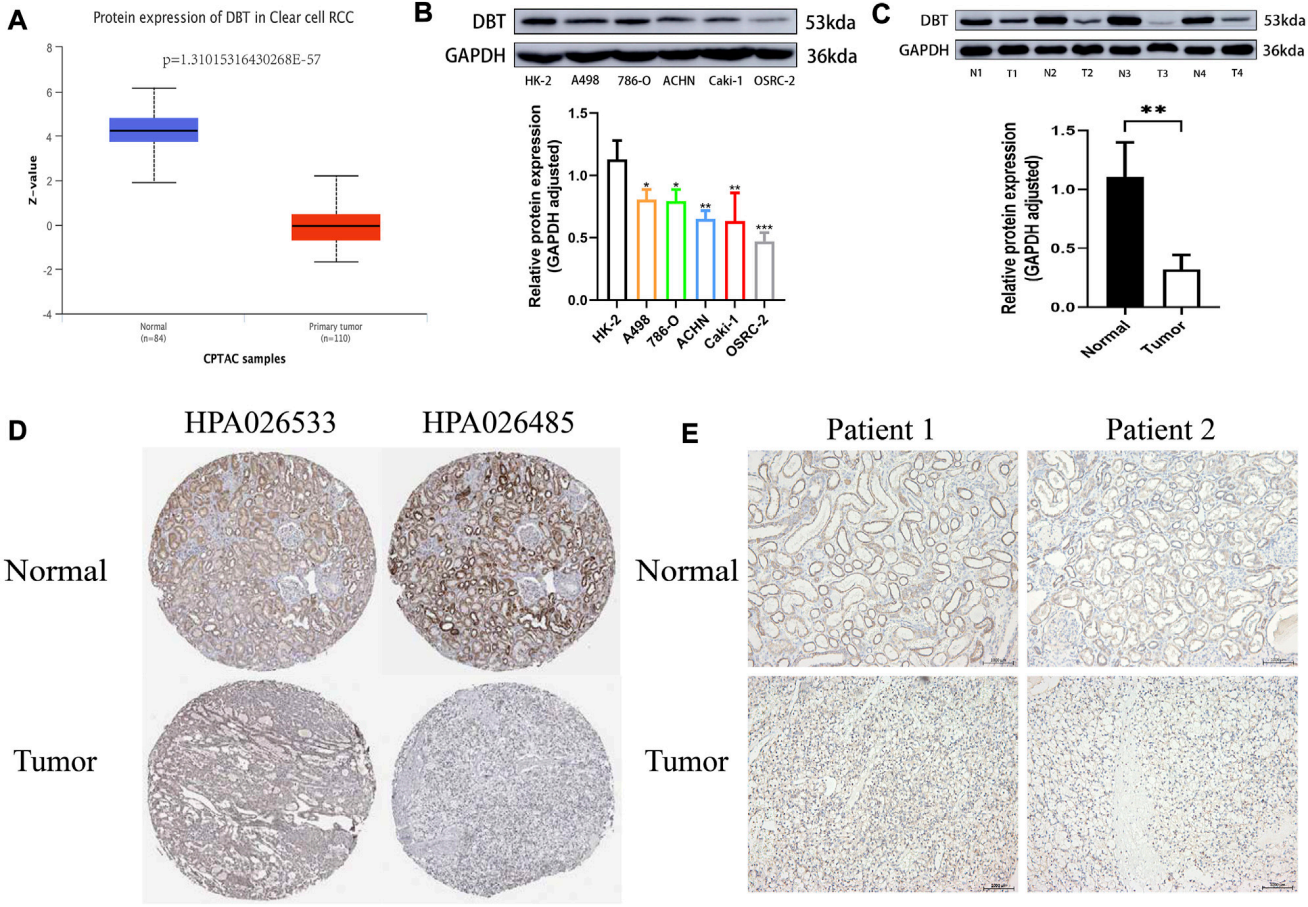
DBT protein expression is low in ccRCC. **(A)** The histogram obtained from the analysis of data from the Clinical Proteomic Tumor Analysis Consortium (CPTAC) on the UALCAN website shows significantly lower expression of the DBT protein in ccRCC tissues versus normal tissues (*p* = 1.31015316430268E-57). **(B)** The results of western blotting of A498, 786-O, ACHN, Caki-1, and OSRC-2 cell lysates showed lower protein expression of DBT in ccRCC cells compared to HK-2 cells, and the histogram quantifying the results also indicated a statistically significant difference. **(C)** Similarly, DBT protein expression was lower in renal clear cell carcinoma tissues than in matched pairs of paraneoplastic normal tissues, and the histogram quantification confirmed this finding. **(D)** Immunohistochemical results from the HPA database showed that DBT expression in ccRCC was mainly in the nucleus, whereas in normal renal tissue, it was in normal tubular epithelial cells. **(E)** This result was also validated by the immunohistochemical results of 2 pairs of ccRCC tissues from our study centre. *p < 0.05, **p < 0.01, ***pp < 0.001, “ns”: not statistically significant,“N”:Normal,“T”:Tumor.

To explore the expression of DBT in ccRCC, the results of TCGA and GEO dataset analysis consistently demonstrated that DBT expression was significantly lower in both renal clear cell carcinoma tissues than in normal tissues (Figure 1A). Similarly, the qPCR results of renal cell carcinoma cell lines (A498, 786-O, ACHN, Caki-1, and OSRC-2) and renal tubular epithelial cells (HK-2), as well as 14 pairs of renal clear cell carcinoma tissues and adjacent normal tissues from our centre, also validated this result (Figures 1B,C). In terms of protein levels, analysis of ccRCC data from the Clinical Proteomic Tumor Analysis Consortium (CPTAC) revealed a significantly lower expression of DBT in cancer tissue compared to normal tissue (*p* = 1.31015316430268E-57) (Figure 2A). Moreover, WB demonstrated significantly lower expression of DBT levels in renal cancer cell lines (A498, 786-O, ACHN, Caki-1, and OSRC-2) than in normal renal tubular epithelial cells (HK-2), and WB of DBT expression in 4 pairs of renal clear cell carcinoma and adjacent normal tissues also showed lower expression in renal clear cell carcinoma tissues(Figures 2B,C). Furthermore, immunohistochemistry was used to analyse DBT expression in the HPA database and 2 pairs of renal clear cell carcinoma tissues and adjacent normal tissues, and the experiments consistently indicated that DBT is mainly distributed in normal renal tubular epithelial cells and in the nucleus of ccRCC cells. (Figures 2D,E).

### Relationship between the mRNA expression level of dihydrolipoamide branched chain transacylase E2 and the clinicopathological features of clear cell renal cell carcinoma patients

**FIGURE 3:**
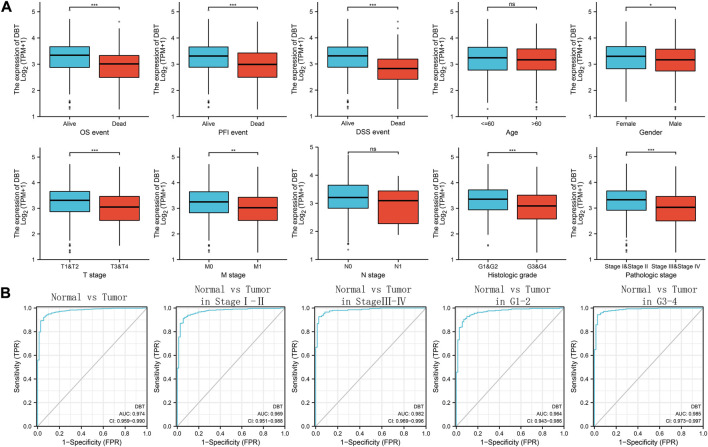
Relationship between DBT expression and clinical features and its diagnostic value. **(A)** Low expression of DBT was significantly related to fatal events in terms of OS, PFI, and DSS, while it was also observed in men and those with higher T stage, M stage, histologic grade and pathologic stage, but there was no significant difference in age or N stage. **(B)** For the diagnostic ability of DBT for ccRCC tumours, the AUC value of DBT for distinguishing ccRCC tissues and normal tissues was 0.974, while the AUC values for distinguishing between different subtypes of ccRCC in pathological stages I-II and III-IV and histological grades 1–2 and 3–4 were 0.974, 0.969, 0.982, 0.964, and 0.985, respectively. *p < 0.05, **p < 0.01, ***p < 0.001, “ns”: Not statistically significant.

The clinical information and DBT expression information of ccRCC patients from TCGA were analysed together. The results showed that DBT expression was low in ccRCC patients with lethal events (including OS, DSS, and PFI) and higher T stage, M stage, histological grade and pathological stage (Figure 3A). ROC curves plotted by R software were used to assess the diagnostic ability of DBT for ccRCC patients with different statuses and showed area under the curve (AUC) values of 0.974 for diagnosing ccRCC and normal tissues. Similarly, DBT expression could better distinguish between different subgroups of ccRCC, and the area under the curve (AUC) values for distinguishing pathological stages Ⅰ-Ⅱ and III-IV and histological grades 1–2 and 3–4 were 0.969, 0.982, 0.964 ,and 0.985, respectively (Figure 3B), indicating that DBT has an extremely strong diagnostic value.

### Prognostic significance of differential dihydrolipoamide branched chain transacylase E2 expression in clear cell renal cell carcinoma patients

**FIGURE 4:**
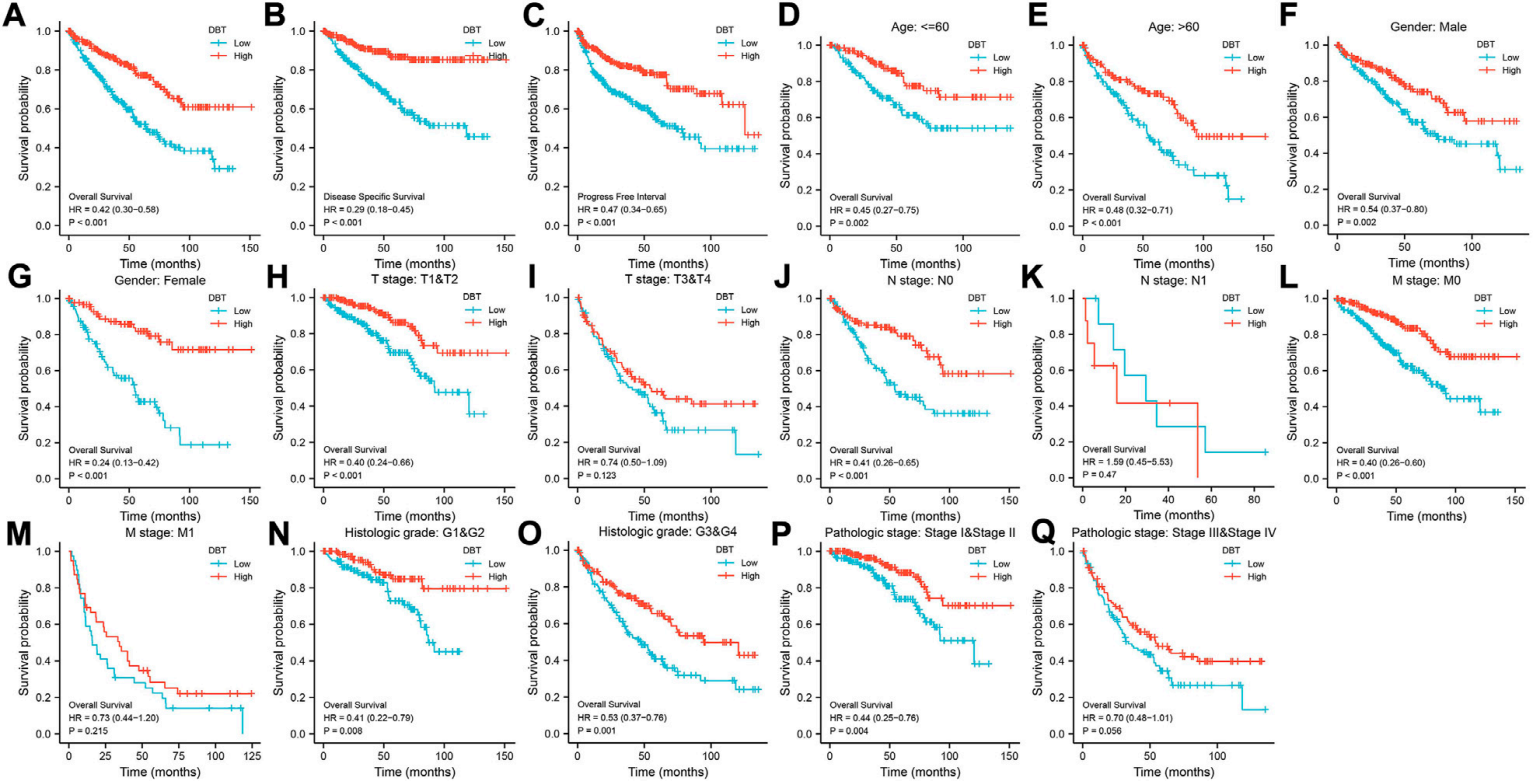
Relationship between DBT expression and the survival of ccRCC patients. In terms of outcomes such as OS **(A)**, DSS **(B)**, and PFI **(C)**, low expression of DBT in ccRCC patients indicated a better prognosis in groups based on age **(D, E)**, male sex **(F)**, female sex **(G)**, T1-T2 stage **(H)**, N0 stage **(J)**, M0 stage **(L)**, M1 stage **(M)**, histologic G1-G2 stage **(N)**, histologic G3-G4 stage **(O)**, and pathologic stages I-II **(P)**. However, there were no significant differences in the prognosis of DBT expression groups for patients with T3-T4 stage **(I)**, N1 stage **(K)**, and pathologic stages III-IV **(Q)**.

**FIGURE 5:**
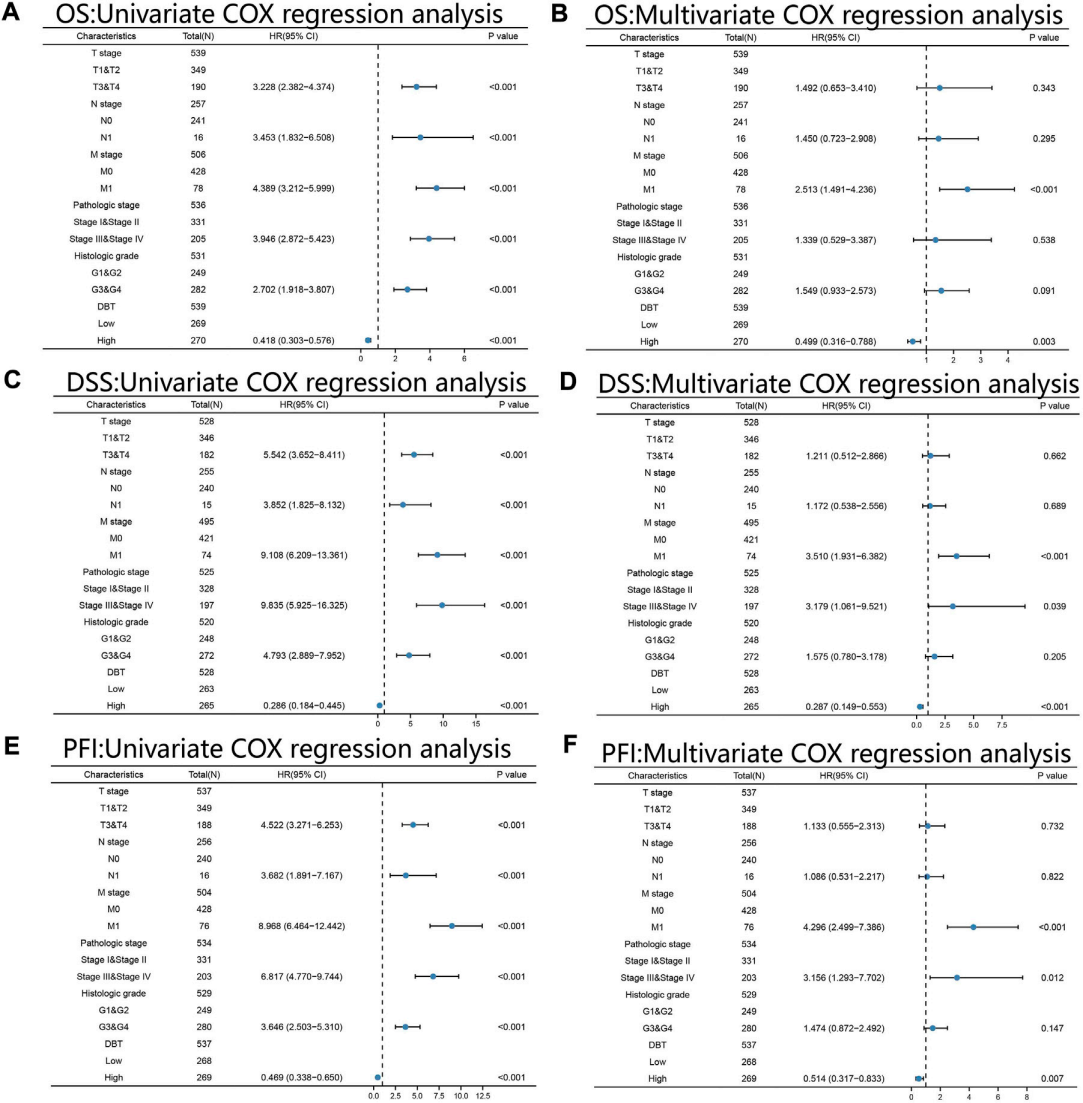
Univariate and multivariate Cox regression analyses. Univariate and multivariate Cox regression analyses of ccRCC based on the expression levels of DBT, T stage, N stage, M stage, pathologic stage, and histologic grade for OS **(A,B)**, DSS **(C,D)** and PFI **(E,F)**. All results consistently showed that DBT alone could be an independent factor in determining the prognosis of ccRCC patients. OS: overall survival; DSS: disease-specific survival; PFI: progression-free interval.

**FIGURE 6:**
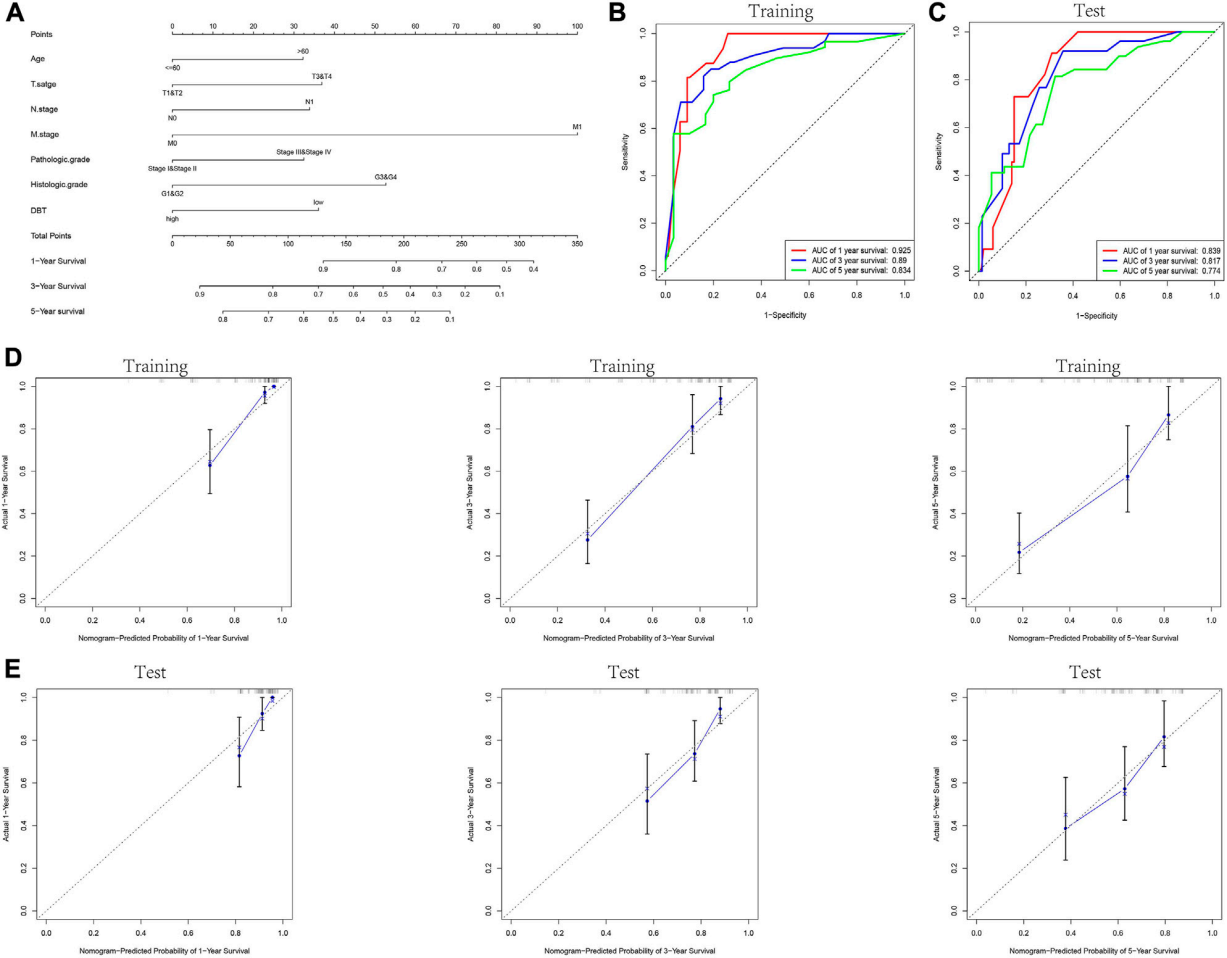
Construction and validation of the nomogram. **(A)** Prognostic nomogram constructed by combining data on clinically relevant factors (e.g., age, T stage, N stage, M stage, pathologic grade and histologic grade) and DBT expression. The TCGA-KIRC dataset was randomly divided into the training and test groups, and the ROC curves for 1, 3, and 5 years were plotted for the training and test groups based on the constructed prognostic judgement model. The AUC values predicting 1-year, 3-year, and 5-year survival were 0.925, 0.89, and 0.834 in the training group **(B)** and 0.839, 0.817, and 0.774 in the test group **(C)**, respectively. **(D,E)** Calibration plots to assess the nomogram demonstrate its predictive power for 1-year, 3-year, and 5-year survival rates in the training and test groups of the TCGA-KIRC dataset.

KM analysis demonstrated the impact of DBT expression on the survival of ccRCC patients in the context of different clinical features. Not surprisingly, patients with low DBT expression had a worse OS, DSS and PFI than patients with high DBT expression (Figures 4A–C). In ccRCC low DBT expression was related to worse OS in patients in both age groups (≤60 vs. >60) and both sex groups and in patients with T stage 1–2, N0, M0, histologic grade G1-2 or G3-4, and pathologic stage I-II, but DBT expression had no significant effect on overall survival in patients with T3-4, N1 or M1 disease or pathologic stage III-IV (Figure 4D-Q). Univariate and multivariate Cox regression analyses were used to further verify whether DBT expression could be an independent prognostic factor, and the univariate Cox regression analysis results showed significant correlations between DBT expression and OS, DSS and PFI in ccRCC patients (all *p* values <0.001) (Figures 5A,C,E). Similarly, the multivariate Cox regression analysis results showed significant correlations between DBT expression and OS, DSS and PFI in ccRCC patients (*p* = 0.003, *p* < 0.001, and *p* = 0.007) (Figures 5B,D,F). We constructed a nomogram based on DBT and several clinical pathological variables (e.g., age, T stage, N stage, M stage, pathological grade, and histological grade), which was used to predict patient survival at 1, 3, and 5 years (Figure 6A). The TCGA-KIRC cohort was randomly divided into training and test groups for mutual validation, where the C-index value for the training group was 0.825 and the AUCs for 1-year, 3-year and 5-year survival were 0.925, 0.89 and 0.834, respectively (Figure 6B). The corresponding indices in the test group were 0.839, 0.817, 0.774, and 0.745 (Figure 6C). The calibration curves of the training and test groups further demonstrated the satisfactory performance of the nomogram (Figures 6D,E). All of the above results consistently indicated that DBT has great potential to become a new biomarker for determining the prognosis of ccRCC patients.

### Construction of protein‒protein interaction networks and exploration of potential mechanisms

**FIGURE 7:**
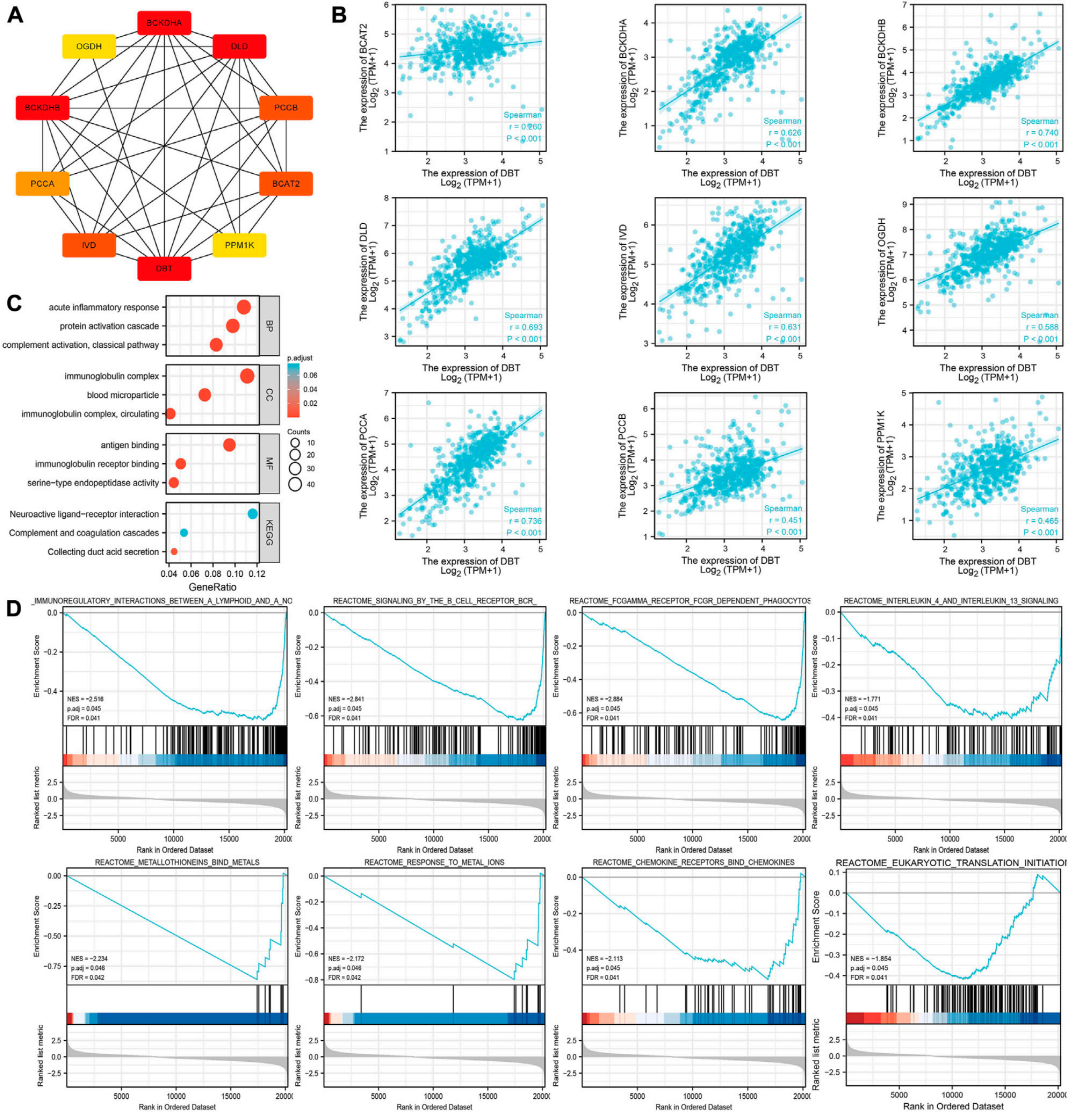
Exploration of the potential functions of DBT in ccRCC. **(A)** The STRING website was used to analyse the DBT-related protein‒protein interaction network, which showed the associations between DBT and BCAT2, BCKDHA, BCKDHB, DLD, IVD, OGDH, PCCA, PCCB, and PPM1K. **(B)** Spearman analysis further demonstrated the associations between these genes, and significant positive associations between DBT and all of them were observed based on the results. **(C)** Gene Ontology (GO) analysis of biological processe (BP), cellular component (CC) and molecular function (MF), and Kyoto Encyclopedia of Genes and Genomes (KEGG) analysis were performed to obtain a comprehensive view of the potential functions of DBT in ccRCC. **(D)** Enrichment plots of DBT expression in terms of immunoregulatory interactions between a lymphoid and a nonlymphoid cell (NES = −2.516, p. adj = 0.045, FDR = 0.041), signalling by the B-cell receptor (NES = −2.841, p. adj = 0.045, FDR = 0.041), FCGAMMA receptor FCGR-dependent phagocytosis (NES = −2.884, p. adj = 0.045, FDR = 0.041), interleukin-4 and interleukin-13 signalling (NES = −1.771, p. adj = 0.045, FDR = 0.041), metallothioneins bind metals (NES = −2.234, p. adj = 0.046, FDR = 0.042), response to metal ions (NES = −2.172, p. adj = 0.046, FDR = 0.042), chemokine receptors bind chemokines (NES = -2.113, p. adj = 0.045, FDR = 0.041), and eukaryotic translation initiation (NES = −1.854, p. adj = 0.045, FDR = 0.041).

It is only by understanding the functional interactions between proteins that we are more likely to be able to understand the mechanisms of cancer development. The PPI network of DBT proteins in ccRCC was consequently analysed by the STRING website, and genes including BCAT2, BCKDHA, BCKDHB, DLD, IVD, OGDH, PCCA, PCCB, and PPM1K were screened (Figure 7A). Spearman analysis showed a significant positive correlation between all these genes and DBT (Figure 7B).

According to GO analysis, DBT was mainly involved in “acute inflammatory response”, “protein activation cascade” and “complement activation, classical pathway” in the BP category; “immunoglobulin complex,” “blood microparticle” and “immunoglobulin complex circulating” in the CC category; and “antigen binding” and “immunoglobulin receptor” in the MF category (Figure 7C). Furthermore, KEGG analysis showed that DBT was mainly involved in “neuroactive ligand‒receptor interaction”, “complement and coagulation cascades” and “collecting duct acid secretion”. GSEA showed that the main pathways enriched in DBT-related genes in ccRCC were immunoregulatory interactions between a lymphoid and a nonlymphoid cell (NES = −2.516, p. adj = 0.045, FDR = 0.041), signalling by the B-cell receptor (NES = −2.841, p. adj = 0.045, FDR = 0.041), FCGAMMA receptor FCGR-dependent phagocytosis (NES = −2.884, p. adj = 0.045, FDR = 0.041), interleukin-4 and interleukin-13 signalling (NES = −1.771, p. adj = 0.045, FDR = 0.041), metallothioneins bind metals (NES = −2.234, p. adj = 0.046, FDR = 0.042), response to metal ions (NES = −2.172, p. adj = 0.046, FDR = 0.042), chemokine receptors bind chemokines (NES = −2.113, p. adj = 0.045, FDR = 0.041), and eukaryotic translation initiation (NES = −1.854, p. adj = 0.045, FDR = 0.041) (Figure 7D). Together, these results suggest that DBT, a gene associated with copper-induced cell death, may have a strong association with immunological effects in the development of ccRCC.

### Correlation analysis of dihydrolipoamide branched chain transacylase E2 expression and immune infiltration

**FIGURE 8:**
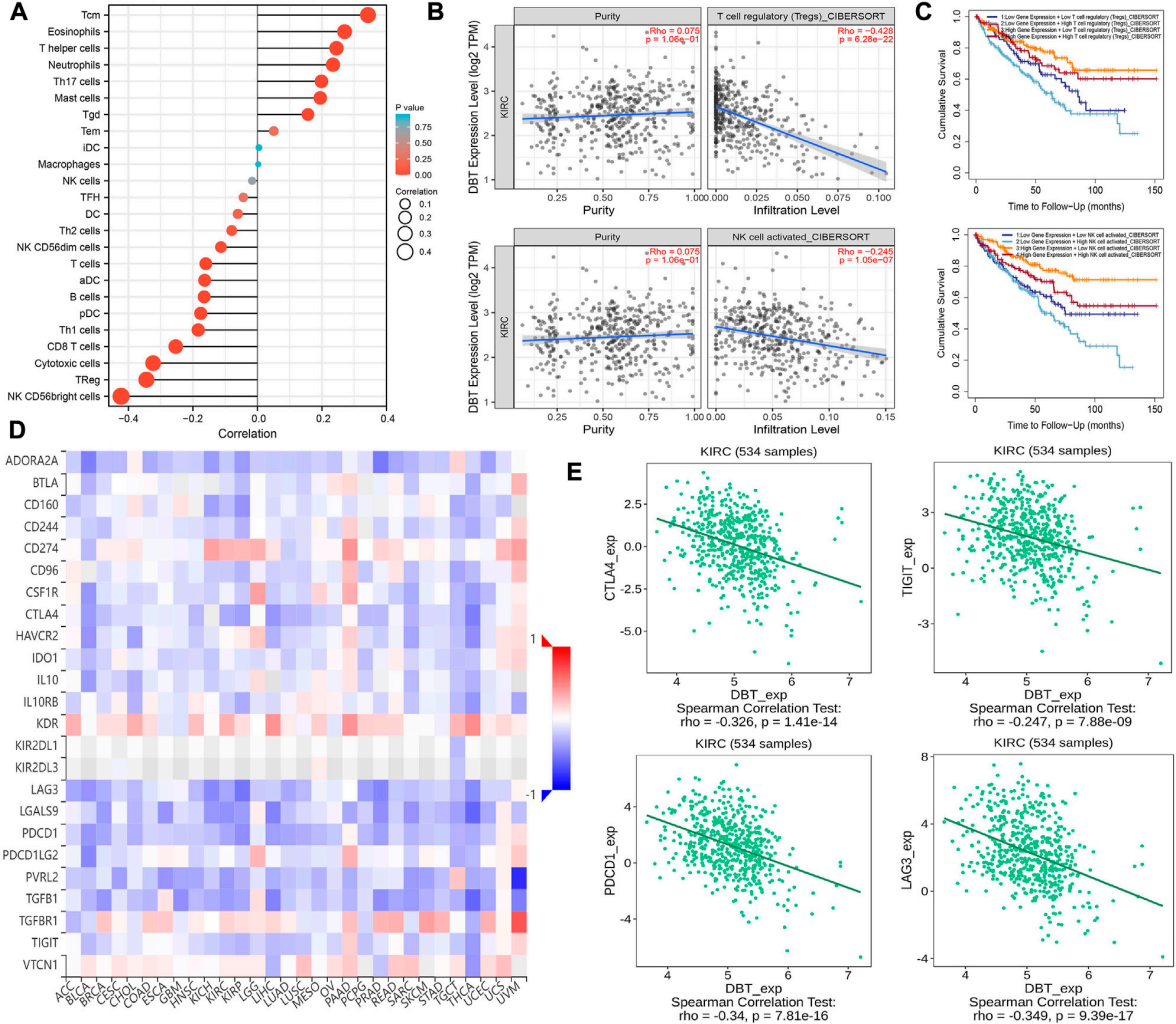
Exploration of the immune characteristics of DBT in ccRCC. **(A)** The correlations between TILs and DBT expression in different immune cells were calculated based on the “GSVA” package, and most of the immune cells were found to have some relationship with DBT expression. **(B)** DBT expression was found to be negatively correlated with the immune infiltration levels of regulatory T cells (Treg) and activated NK cells through the TIMER2.0 website based on the “CIBERSORT” algorithm. **(C)** Similarly, DBT gene expression was analysed in relation to Treg and activated NK-cell immune infiltration levels for the survival of ccRCC patients. **(D)** The relevance of immunosuppression across cancers. **(E)** There were significant negative correlations between DBT expression and immune checkpoint genes (CTLA4, TIGIT, LAG3, and PDCD1) in ccRCC.

The single-sample GSEA (ssGSEA) algorithm of the “GSVA” R software package revealed that DBT related to the majority of TILs in ccRCC; for example, positive correlations were found between DBT expression and the infiltration of Tcm cells, eosinophils, T helper cells, neutrophils, Th17 cells, and mast cells, and negative correlations were found between DBT expression and the infiltration of NK CD56dim cells, T cells, aDCs, B cells, pDCs, Th1 cells, CD8 T cells, cytotoxic cells, Treg cells, and NK CD56bright cells, while there was no significant association with Tgd, Tem cells, iDCs, macrophages, NK cells, TFH cells, and DCs (Figure 8A). Moreover, the CIBERSORT algorithm provided on the TIMER 2.0 website was used to further calculate the correlations between DBT and the infiltration levels of Tregs and activated NK cells in ccRCC and to perform survival analysis under different states. The results showed that DBT expression in ccRCC was significantly and negatively correlated with the infiltration of Tregs (R = −0.428, *p* = 6.28e-22) and NK CD56bright cells (R = −0.245, *p* = 1.05e-07) (Figure 8B). Survival analysis showed that the cumulative survival time of ccRCC patients with low expression of DBT and high infiltration of Tregs or activated NK cells was less than that of patients with high expression of DBT with low infiltration of Tregs or activated NK cells (Figure 8C). The GEPIA and TIMER databases were used to jointly analyse the relationship between DBT and some markers of corresponding immune cells, which showed that most marker of immune cells in ccRCC were associated with DBT expression (Supplementary Table S1).

In addition, the internal relationship between DBT expression in ccRCC and immune checkpoint genes was investigated on the TISIDB website, and a clear negative correlation was found between DBT and immunoinhibitors, such as CTLA4 (r = −0.326, *p* = 1.41e-14), TIGIT (r = −0.247, *p* = 7.88e-09), LAG3 (r = −0.349, *p* = 9.39e-17), and PDCD1 (r = −0.34, *p* = 7.81e-16) (Figures 8D,E).

## Discussion

ccRCC is a heterogeneous disease with high rates of tumour recurrence and metastasis, which often predict a very poor clinical outcome. ccRCC is also highly infiltrated with immune cells, and thus, immunotherapy has a leading role in its systemic treatment. Despite the heterogeneity of ccRCC and the complex molecular regulatory network of the tumour microenvironment (TME), these therapeutic strategies still hold great promise (Krishna et al., 2021). Here, we explored the expression and prognostic significance of DBT in ccRCC and identified DBT as a tumour suppressor gene.

The current study found for the first time that both the mRNA expression and protein expression of DBT were downregulated in ccRCC, and were also verified in many renal cancer cell lines and in renal clear cell carcinoma tissues from our study centre. After further analysis, we found that its expression was negatively correlated with the occurrence of fatal events in terms of OS, DSS, and PFI, high T and M stages, high histologic grade and high pathologic stage. The ROC curve analysis results showed that DBT had high AUC values in distinguishing between different states of ccRCC and the normal kidney state, and these results indicate that DBT has high potential to become a biomarker for the diagnosis of ccRCC. Both KM analysis and univariate and multivariate Cox regression analyses consistently demonstrated that low DBT expression in ccRCC was strongly associated with poor patient prognosis. The construction of a nomogram allowed us to predict the probability of OS at 1, 3 and 5 years by combining multiple indicators (age, T stage, N stage, M stage, pathologic grade, histologic grade, and DBT expression) for the quantitative scoring of ccRCC patients, while TCGA-KIRC data grouping, the C-index, ROC curves and calibration curves confirmed the high accuracy of the quantitative model. Based on these findings, we collectively concluded that there is great potential for DBT to serve as a prospective diagnostic and prognostic biomarker.

DBT is mainly involved in signalling pathways related to the immune response in ccRCC; thus, the correlation between DBT and the immunological profile of ccRCC was further explored. The TME, consisting of tumour cells, TILs, stromal cells and extracellular matrix, is closely related to tumorigenesis and progression (Pitt et al., 2016; Hinshaw and Shevde, 2019). Treg cells, a type of immunosuppressive cell, have been shown to cause the immune escape of tumour cells in ccRCC, thereby contributing to tumour cell proliferation and metastasis and ultimately to a poor prognosis (Pottier et al., 2015; Tada et al., 2018; Kim et al., 2020; Xiong et al., 2020). Interestingly, a negative correlation between DBT expression and Treg cells was found in ccRCC, and the poor prognosis of patients with low DBT expression and high Treg cell infiltration was verified to be more prominent based on survival analysis.

NK cells can directly and rapidly kill tumour cells by receptor recognition, and there are different subtypes of cells with high heterogeneity (Hodgins et al., 2019; Wu et al., 2020). The activity of NK cells and their degree of tumour infiltration are closely related to prognosis, and NK-cell infiltration to sites of lung metastases in ccRCC is beneficial to improve survival (Winter et al., 1992; Schleypen et al., 2003; Remark et al., 2013). However, the activation of NK cells is often determined by the balance between activating and inhibiting signals emitted by some receptors in combination with the corresponding ligands, and NK cells respond only when the activating signal is stronger than the inhibiting signal (Cerboni et al., 2007; Ardolino et al., 2011; Hodgins et al., 2019). Notably, the performance of activated NK cells in ccRCC in this study showed a diametrically opposite trend to that in previous studies. This is because on the basis of the negative correlation between activated NK cells and DBT expression, the OS of ccRCC patients decreased significantly with the downregulation of DBT expression and the enrichment of activated NK cells. In contrast, when DBT expression was upregulated and activated NK cells were depleted, the prognosis of patients was greatly improved. Intriguingly, not only are new findings described here, but the tumour suppressive effects of DBT and its crosstalk with TILs are also debated.

Currently, immunotherapy for tumours focuses on the restoration of the host’s antitumour immune response by blocking immune checkpoints (Sanmamed and Chen, 2018). Blockade strategies targeting the immune checkpoint genes CTLA4 (Topalian et al., 2016; Atkins and Tannir, 2018; Braun et al., 2021), TIGIT (Hong et al., 2018; Takamatsu et al., 2021), LAG3 (Klümper et al., 2020), and PDCD1 (Sunshine and Taube, 2015; Hayashi and Nakagawa, 2020) have long been or will soon be effective tools for the treatment of advanced ccRCC. However, DBT was significantly negatively correlated with these molecules, implying that DBT expression might counteract immune escape or immunosuppression in ccRCC to some extent.

Although this study illustrates for the first time the function and potential mechanism of DBT in ccRCC, it still has some limitations. More comprehensive, relevant and valid sample data need to be collected and analysed to increase the reliability of the results. Moreover, further experimental studies are needed to reveal the specific mechanism by which DBT plays a role in ccRCC.

## Conclusion

In summary, our study showed that DBT expression was lower in ccRCC tissue than in normal tissue, and indicated a poor prognosis. Moreover, DBT has great potential to become a new biomarker for ccRCC diagnosis and prognosis evaluation. Furthermore, we found that DBT is negatively associated with immunosuppressive features in ccRCC, which may provide new ideas for immune checkpoint inhibitor-based therapy.

## Data Availability

Publicly available datasets were analyzed in this study. This data can be found here: TCGA database (TCGA-KIRC) and GEO database (accession number: GSE53757, GSE53000, GSE40435, and GSE105261).Code is visible in the public repository (https://github.com/xiping0723/Rowdata.git).
